# Influence and Mechanisms of Action of Environmental Stimuli on Work Near and Above the Severe Domain Boundary (Critical Power)

**DOI:** 10.1186/s40798-022-00430-1

**Published:** 2022-03-28

**Authors:** Normand A. Richard, Michael S. Koehle

**Affiliations:** 1 Richard Physiological Services, Port Moody, Canada; 2 School of Kinesiology, University of British Columbia, Vancouver, Canada; 3 Division of Sports Medicine, University of British Columbia, Vancouver, Canada

**Keywords:** Critical power, Severe domain, Exercise, Hypoxia, Heat, Cold, Air pollution

## Abstract

**Abstract:**

The critical power (CP) concept represents the uppermost rate of steady state aerobic metabolism during work. Work above CP is limited by a fixed capacity (*W*′) with exercise intensity being an accelerant of its depletion rate. Exercise at CP is a considerable insult to homeostasis and any work done above it will rapidly become intolerable. Humans live and exercise in situations of hypoxia, heat, cold and air pollution all of which impose a new environmental stress in addition to that of exercise. Hypoxia disrupts the oxygen cascade and consequently aerobic energy production, whereas heat impacts the circulatory system’s ability to solely support exercise performance. Cold lowers efficiency and increases the metabolic cost of exercise, whereas air pollution negatively impacts the respiratory system. This review will examine the effects imposed by environmental conditions on CP and *W*′ and describe the key physiological mechanisms which are affected by the environment.

**Graphical Abstract:**

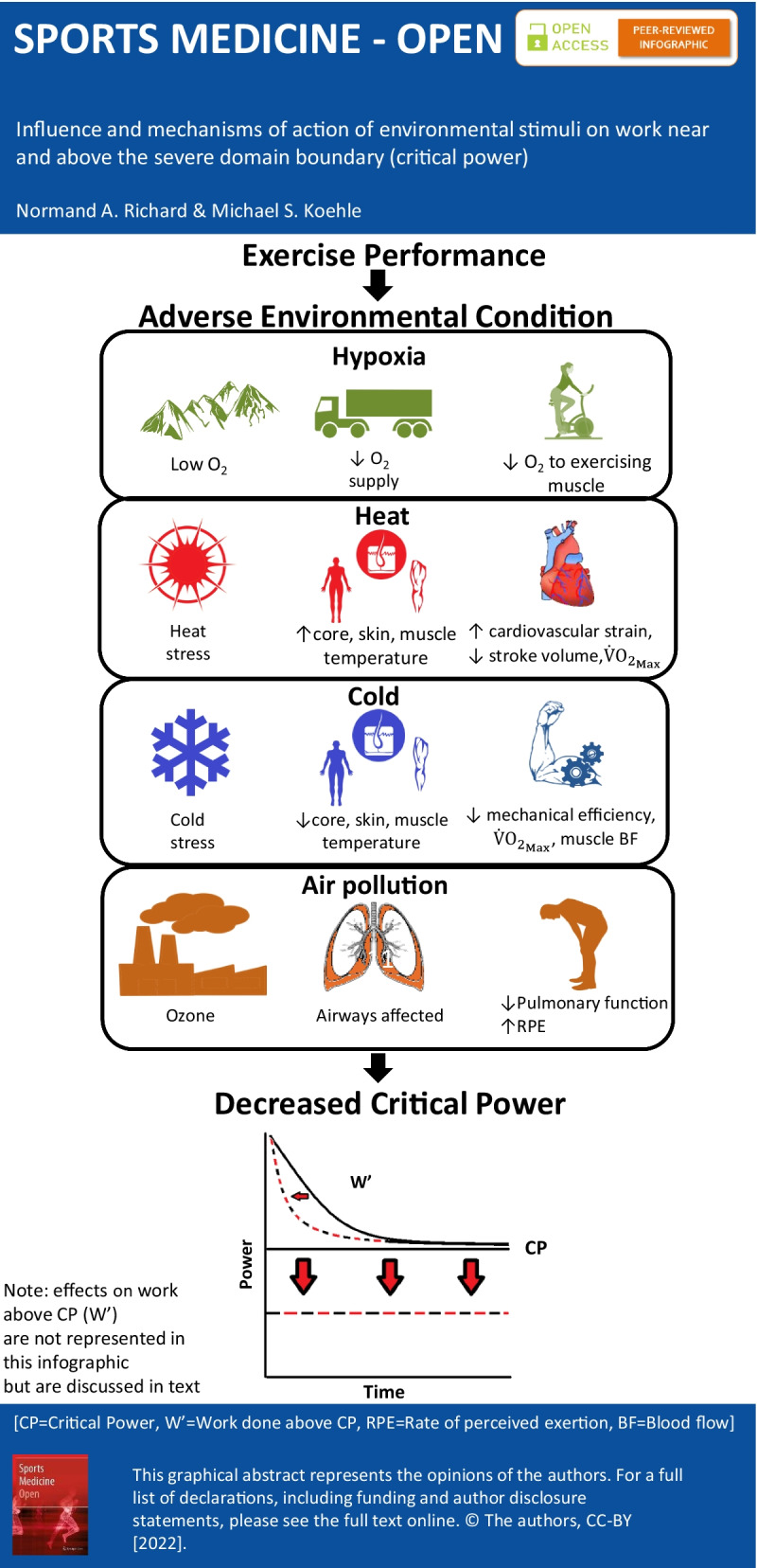

## Key Points

- Critical power (CP) represents the highest rate of steady state aerobic metabolism; work capacity above CP is finite and is called *W*′.
- Hypoxia challenges the oxygen delivery cascade, thus lowering CP and (if severe enough) *W*′ as well.
- Temperature perturbations chiefly affect CP by lowering or increasing body temperature thus disturbing homeostasis
- Exercise in air pollution, especially ozone, may lower CP through an effect on airways.

## Introduction

Oxygen travels via the oxygen cascade, from the atmosphere to the mitochondrion by means of partial pressure gradients. Humans are continuously consuming oxygen in proportion to work intensity. Oxygen consumption at rest (${\dot{\text{V}}\text{O}}_{2}$) is ~ 3.5 ml kg^−1^ min^−1^. During rhythmic large muscle mass exercise it may reach remarkable maximal values of ~ 70 and 80 ml kg^−1^ min^−1^ in highly trained women and men, respectively.

**Fig. 1:**
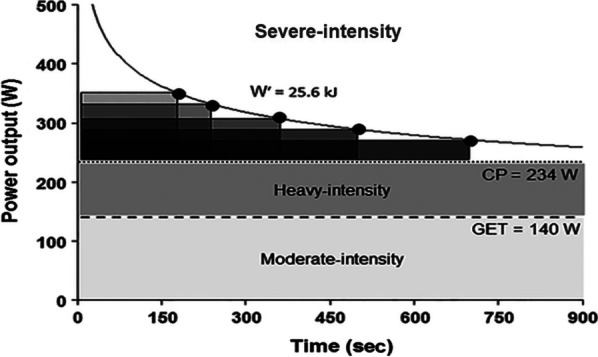
Visual representation of exercise intensity domains. The moderate intensity domain is hallmarked by achievement of steady-state ${\dot{\text{V}}\text{O}}_{2}$, and minimal [Lactate] or [K^+^] accumulation occurs. It is capped by the gas exchange threshold (GET). Hallmark features of the heavy domain include a delay in achieving a steady-state ${\dot{\text{V}}\text{O}}_{2}$ and a rise but stabilisation of [Lactate]; its upper boundary is CP. The severe domain encompasses all work done above CP, and is limited by a finite capacity (*W*′). ${\dot{\text{V}}\text{O}}_{2}$ and [Lactate] kinetics do not reach steady state and muscle [PCr] and pH reach low values [5]. Figure adapted from Jones et al. [11] and reused under Creative Commons License

Conceptually, critical power (CP) represents the maximal rate of aerobic steady state work and demarcates the heavy and severe exercise domains [1]. CP varies with health and training status. In chronic heart failure, patient CP is 65% of peak power achieved during an incremental exercise test [2]. By contrast, in healthy, recreationally active young individuals, CP can reach 70–80% of ${\dot{\text{V}}\text{O}}_{{2_{{{\text{Max}}}} }}$ [3, 4], while highly aerobically trained athletes have a CP near 80–90% of ${\dot{\text{V}}\text{O}}_{{2_{{{\text{Max}}}} }}$ [1]. For example, in professional cyclists who have previously won a World Championship (track endurance) CP has been determined to be 80–85% of peak power achieved during an incremental exercise test (NR personal communication). Work above CP (termed the severe domain) has a finite timeframe with intensity being inversely correlated to duration (Fig. 1). ${\dot{\text{V}}\text{O}}_{2}$ does not reach steady state above CP; it reaches a peak or a maximum alongside plasma [K^+^] and [lactate] accumulation [5]. Work below CP involves achieving a “steady state” of some sort; i.e., where metabolic pathways meet the energetic demands. Although not indefinite, exercise time ranges from many minutes to hours with failure due to limitations such as substrate availability (e.g. glycogen) or cardiac drift (see heavy and moderate domains in Fig. 1) [5, 6]. The heavy and moderate domains are separated by the gas exchange threshold (GET); the breakpoint in the linear rise of ${\dot{\text{V}}\text{O}}_{2}$ and ${\dot{\text{V}}\text{CO}}_{2}$. Regarding units, CP is expressed in watts and the finite capacity (*W*′) in Joules. High values for CP are associated with increased skeletal muscle capillarization and the proportion of type 1 muscle fibres while *W*′ is correlated with thigh volume [7, 8]. The reader is directed to the excellent review of Poole et al. [1] for in-depth discussions of CP. It is worth highlighting that the methodology used (i.e. mathematical equation, exercise trial length) for determining CP may influence the end value; this is fully discussed here [9]. It is remarkable that humans live at altitude, in hot and cold climates, and situations of heavy air pollution. Furthermore, for sporting or military purposes humans attempt to perform near CP in such environments, imposing additional challenges to the already perturbed homeostasis of exercise. We have chosen to limit this review to exercise in the severe domain and above, and to exclude the moderate domain to solely focus on CP and *W*′ as this represents the upper limit of human work which is applicable to numerous speed-based athletic endeavours (i.e. track and field, swimming, track cycling, etc.). In some instances, work near the upper limits of the heavy domain will be included as this would relate to exercise near/slightly below CP. A practical example of this being a report by Jones and Vanhatalo who demonstrated that elite runners complete the marathon distance at 96% of their critical speed (CS) (group mean), in other words essentially at the severe domain boundary [10]. Critical speed represents the running equivalent for CP in cycling. CS is measured in m s^−1^ and *D*’ is the distance travelled (m) above critical speed [1].

As such, this narrative review will explore how environmental stressors influence work at the severe domain boundary and above, focusing on key physiological mechanisms.

## Influence of Environmental Perturbations

This section will examine environmental influences on work at and above CP and the underpinning physiological processes. Whilst numerous environmental conditions may coexist (e.g. cold and altitude), each will be examined individually focusing on acute effects. For brevity, acclimation will not be discussed and hyperbaria and microgravity will be omitted due to their reliance on technological assistance.

Research on CP in environmental physiology is recent, and thus many studies in this review do not *directly* examine CP and *W*′. Regardless, we can make inferences based on duration and intensity of the exercise involved. Therefore, time to exhaustion (TTE), efforts near lactate threshold, GET or 70–90% of ${\dot{\text{V}}\text{O}}_{{2_{{{\text{Max}}}} }}$ are indicative of effects on CP and studies with a sprint/anaerobic component estimate effects on *W*′. We acknowledge this being a necessary, but manageable limitation.

### Hypoxia

Hypoxia imposes a lower ambient partial pressure of inspired O_2_ ($P_{{{\text{I}}_{{{\text{O}}_{2} }} }}$), translating to lower oxygen availability to bodily tissues and ultimately the mitochondria. Given that CP represents the highest rate of steady state aerobic metabolism, impaired oxygen delivery lowers this upper ceiling of steady state work. Hypoxia is induced by reducing barometric pressure ($P_{{\text{B}}}$) or the fraction of inspired oxygen ($F_{{{\text{I}}_{{{\text{O}}_{2} }} }}$). At high-altitude, diminished $P_{{\text{B}}}$ reduces partial pressure of gases and consequently oxygen availability to tissues. Additionally, lower gas density diminishes resistance to movement: this being of significant importance in fixed distance contests. For instance, the current one-hour cycling record (55.089 km) was established at an elevation of 1800 m, surpassing the previous record set at 563 m [12] with a model estimating a 1.68 km h^−1^ advantage when riding at 2338 m and a 1.58 km h^−1^ advantage at 1829 m both compared to sea level [13]. The reader may further examine the effect of hypobaria in the context of hypoxia here [14]. However, the remainder of this section will solely focus on the effects of hypoxia since they have been directly studied in the context of CP. Acute moderate hypoxia ($F_{{{\text{I}}_{{{\text{O}}_{2} }} }}$ 0.15) has been shown to cause a 14% reduction in CP but no change in *W*′ [15]. Within the same study, resting and end-exercise blood oxygen saturation (${\text{Sp}}_{{{\text{O}}_{2} }}$),${\dot{\text{V}}\text{O}}_{{2_{{{\text{Peak}}}} }}$, and power output during ergometer trials designed to elicit fatigue in ~ 3 and ~ 15 min were all lower during exercise in acute hypoxia. Similar results are seen in trained cyclists; CP decreased (270 ± 49 to 225 ± 35 watts) in acute hypoxia ($F_{{{\text{I}}_{{{\text{O}}_{2} }} }}$ 0.13) yet *W*′ remained unchanged [16]. Correspondingly, in recreationally active females, CP is reduced (175 ± 25 to 132 ± 17 watts) in acute hypoxia ($F_{{{\text{I}}_{{{\text{O}}_{2} }} }}$ 0.13) with *W*′ remaining stable [17]. However, decrements in CP are seen during acute hypoxic arm cycling in men [18] but not women [19]. Differences in muscle mass or vasodilatory response could explain these differences [19]. Sex-based variations in lean mass exist, with women having lower distribution of their muscle mass in their upper torso. Absolute strength also differs but when reported relative to lean mass, the discrepancy is superior in upper limbs [20]. Lower absolute CP (CP normoxia 90 vs. CP hypoxia 85 watts in men [18] and CP normoxia 57 vs. CP hypoxia 56 watts in women [19]) in part due to less muscle mass may have obscured a difference between sexes. Young women also show a superior vasodilatory response to an acute hypoxic stimulus during forearm exercise with heightened β-adrenergic receptor activation being a potential mechanism [21]. Overall, these findings are not surprising given our knowledge of performance and ${\dot{\text{V}}\text{O}}_{{2_{{{\text{Max}}}} }}$ decrements with hypoxia [22]. Briefly,${\dot{\text{V}}\text{O}}_{{2_{{{\text{Max}}}} }}$ decreases in a curvilinear fashion with increasing hypoxia with limited oxygen pulmonary diffusion (which increases the alveolar to arterial $P_{{{\text{O}}_{2} }}$) playing a major role [23, 24]. Work done above CP rapidly approaches ${\dot{\text{V}}\text{O}}_{{2_{{{\text{Max}}}} }}$, (i.e. ${\dot{\text{V}}\text{O}}_{2}$ is brought rapidly to the maximum). Further, we know that acute hypoxia reduces ${\dot{\text{V}}\text{O}}_{{2_{{{\text{Max}}}} }}$, by means of lowered $P_{{{\text{I}}_{{{\text{O}}_{2} }} }}$, lung gas exchange, maximal cardiac output (CO) and peak leg blood flow [25] and it is thus not surprising that CP is decreased in hypoxia.

**Fig. 2:**
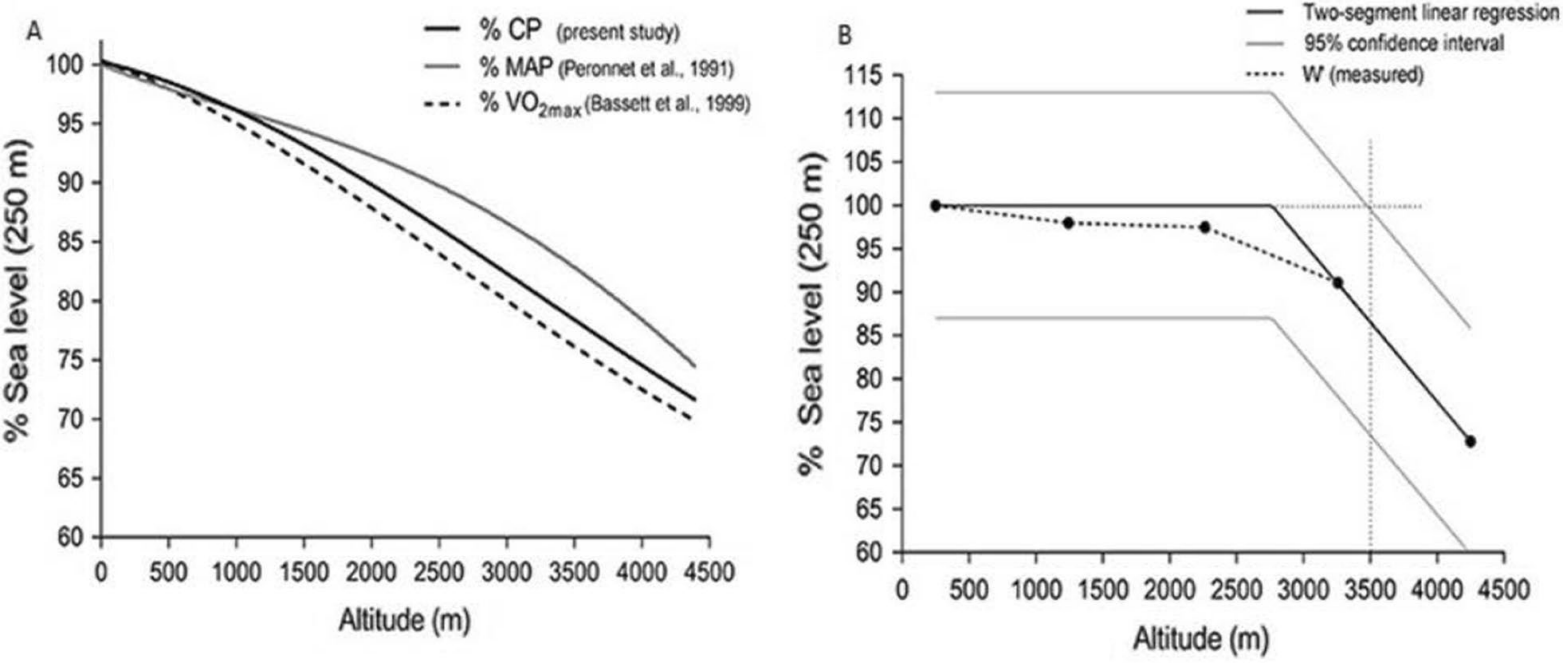
Panel A illustrates the predictive model of CP decrease in acute hypoxia exposures alongside previously reported decrements in maximal aerobic power (MAP) and ${\dot{\text{V}}\text{O}}_{{2_{{{\text{Max}}}} }}$. Panel B shows the decrease in *W*′ which appears unaffected until 4,250 m ($F_{{{\text{I}}_{{{\text{O}}_{2} }} }}$ 0.123). Figure adapted from [28] and reused under Creative Commons License

It was previously thought that *W*′ represented an “anaerobic energy store” independent of oxygen. We now know that this is more complex than initially conceived, as hypoxia and hyperoxia manipulations affect *W*′ [26]. For example, testing in severe hypobaric hypoxia ($F_{{{\text{I}}_{{{\text{O}}_{2} }} }}$ ~ 0.105) achieved over a two-week gradual ascent not only lowers CP (123 ± 38 vs. 81 ± 21 W), but it also decreases *W*′ (13.1 ± 4.3 vs. 7.2 ± 2.9 kJ) [27]. Explaining the rationale for the decreased *W*′ is less clear than for lowered CP. A likely mechanism is reduced muscle and venous oxygen concentration which would lower the amount of “ready-to-use” oxygen stores available for work above CP [27]. Valli et al. [27] also discuss blood flow being redistributed towards the respiratory muscles to the detriment of the exercising skeletal muscle and increased dyspnea resulting from the pronounced exercise ventilation rates (${\dot{\text{V}}}_{{\text{E}}}$) at altitude, which suggests that perhaps ventilation limitation also contributes as a limiting factor to work done above CP. Supporting the above, using experimental and modeling approaches, a curvilinear reduction in CP occurs with increasing altitude, whereas *W*′ only markedly decreases at an $F_{{{\text{I}}_{{{\text{O}}_{2} }} }}$ of 0.123 which corresponds to an altitude of 4250 m in Fig. 2 [28]. Further to decreased muscular oxygen availability, Townsend et al. [28], propose lowered central motor drive, resulting from hypoxia, could be responsible for the *W*′ reduction. However, there is one possible countermeasure to this hypoxic effect on *W*′. One study has shown that ergogenic interventions such as NaHCO_3_ augment *W*′ in both normoxia and hypoxia ($F_{{{\text{I}}_{{{\text{O}}_{2} }} }}$ 0.145) while CP is not affected. This enhanced capacity for work above CP appears to stem from increased intramuscular H^+^ shuttling and higher rates of glycolytic flux which benefit from an alkaline environment [29]. Supplementation with nitrates may also be of benefit especially at higher intensities when more type II fibres are recruited [30]; the benefits for nitrate supplementation at altitude and in general have been previously reviewed [31, 32]. However, this is not a consistent finding. In trained runners 90 s interval sessions at 110% of peak running speed in acute hypoxia are not improved by nitrate supplementation [33]. In summary, evidence is clear that acute hypoxia lowers CP, yet only severe hypoxia affects *W*′.

### Heat

Given that human movement generates considerable heat, it is remarkable that homeostasis maintains core temperature ($T_{{{\text{Core}}}}$) near 37 °C so effectively. In hot environments, heat dissipation is challenging during exercise because of a narrowing of the temperature gradient between the body and its environment, with evaporation of sweat becoming the principal mechanism for heat loss.

This section will examine studies which show impaired aerobic exercise performance in heat. To begin, TTE at 70% ${\dot{\text{V}}\text{O}}_{{2_{{{\text{Max}}}} }}$, lasts ~ 52 min in 31 °C versus 94 min in 11 °C, demonstrating a detrimental effect of heat on exercise near CP [34]. Numerous well-controlled studies using self-paced time trials in trained cyclists have examined the effects of heat (35 °C) versus a neutral condition on performance, where in the neutral condition cyclists worked at ~ 80–85% of ${\dot{\text{V}}\text{O}}_{{2_{{{\text{Max}}}} }}$ or at CP. A common observation is that power output and ${\dot{\text{V}}\text{O}}_{2}$ are generally not different or close to control for the first 10–15 min before decreasing, indicating that an increase in $T_{{{\text{Core}}}}$ is necessary before decrements are observed despite skin temperature being elevated [35–37]. In these studies, the circulatory system experiences elevated heart rate (HR), decreased stroke volume, attenuated cardiac output and mean arterial pressure which ultimately leads to lower ${\dot{\text{V}}\text{O}}_{2}$ and end-of-effort ${\dot{\text{V}}\text{O}}_{{2_{{{\text{Peak}}}} }}$. In addition, rate of perceived exertion and thermal comfort are repeatably higher in hot versus neutral conditions highlighting the potential influence of conscious regulatory processes [35–38].

${\dot{\text{V}}\text{O}}_{{2_{{{\text{Max}}}} }}$ is generally unaffected by heat over a short exposure time (< 15 min) in the absence of preheating in both women and in men [37, 39, 40]. In this scenario, cardiac output is maintained by reallocation of blood to the skeletal muscle via select vasoconstriction (i.e. hepatic) [38, 41]. Increased skin temperature alone does not impair ${\dot{\text{V}}\text{O}}_{{2_{{{\text{Max}}}} }}$: an elevation in $T_{{{\text{Core}}}}$ is also required as shown by a study using water perfused suits to heat cutaneous vessels alone or to heat both the periphery and raise $T_{{{\text{Core}}}}$ concomitantly [42]. Further, early reports show that work time in 40 °C at a workload equivalent to that achieved at ${\dot{\text{V}}\text{O}}_{{2_{{{\text{Max}}}} }}$ is decreased by ~ 25% only when pre-heating occurs, thus truly highlighting the effects of raised body temperature [43].

In trained cyclists, time to fatigue at a constant work rate of 80% peak power output is decreased (7.6–5.4 min) and ${\dot{\text{V}}\text{O}}_{{2_{{{\text{Max}}}} }}$ lowered (4.72–4.28 L min^−1^) after increasing skin temperature by 10 °C and $T_{{{\text{Core}}}}$ by 1 °C [44]. Here, heat stress lowers ${\dot{\text{V}}\text{O}}_{{2_{{{\text{Max}}}} }}$, by accelerating the decrease in cardiac output and mean arterial pressure, leading to diminished exercising muscle blood flow and consequently oxygen delivery compared to control [44]. It is worth noting that the accelerated detrimental effects in this study were exacerbated by both peripheral and core pre-heating which was absent in the abovementioned studies.

A key contributor to this decreased ${\dot{\text{V}}\text{O}}_{{2_{{{\text{Max}}}} }}$, cardiac output, and work above CP, is the increased maximal heart rate (${\text{HR}}_{{{\text{Max}}}}$) seen in hyperthermia (via increases in sympathetic activity and/or action on the sinoatrial node) which reduces filling time and lowers stroke volume [35, 38, 44–46]. Lowered central blood volume reduces cardiac filling pressures, in conjunction with less time for diastolic filling [46] leading to the reduced stroke volume. As such, the reduced arterial blood delivery has its greatest effect during severe exercise when the affected cardiac output cannot meet the working muscles’ demands [46].

With increased $T_{{{\text{Core}}}}$, skin blood flow increases to offload metabolic heat from the core to the periphery and into the environment [47], but while exercising in heat, skin blood flow does not reach its “true” maximum as in resting conditions; it flattens-off when $T_{{{\text{Core}}}}$ reaches ~ 38 °C [48]. Additionally, prolonged exercise in the heat leads to dehydration affecting blood volume and ultimately stroke volume [48]. The central nervous system role is mostly involved during longer exercise when $T_{{{\text{Core}}}}$ and/or brain temperature near ~ 40 °C by decreasing motor activation [46].

Short sequences of intense efforts above CP are not influenced by heat. For instance, two 3 × 30 s sprint sessions separated by an hour, in 40 °C showed no difference in power output to those performed in 22 °C. However, pre-loading sprint efforts with exercise in the heat affects performance. Forty minutes of intermittent exercise in 40 °C at 60% ${\dot{\text{V}}\text{O}}_{{2_{{{\text{Peak}}}} }}$ impairs a set of 5 × 15 s sprints as opposed to control [49]. The increase in muscle temperature during heat exposure benefits individual sprint performance and to some extent repeated sprint efforts, yet as stated by Girard et al. [50], there will be a tipping point when the benefits of heat on repeated sprints will be outweighed by the metabolic, cardiovascular strain, and lowered voluntary muscle activation caused by heat. From the above section, the insult appears ultimately to be the combined increases in core, skin, and muscle temperature which challenge the concomitant demands of heat dissipation and severe exercise. In summary, when sufficient exposure time has raised core temperature or if pre-heating occurs exercise near/at CP becomes affected. The evidence from the sprint and supramaximal studies indicate that *W*′ likely remains unaffected during a brisk exposure to heat.

### Cold

Exercise below the severe domain boundary is affected by cold stress. Subjects performing cycling TTE (70%${\dot{\text{V}}\text{O}}_{{2_{{{\text{Max}}}} }}$) in 4, 11, 21, and 31 °C (without pre-cooling) show a U-shaped performance relationship [34]. TTE is longest in 11 °C (93.5 min), and shortest in 31 °C (51.5 min) with 4 °C and 21 °C having no difference in times (~ 81 min). Cardiorespiratory variables (measured every 15 min) show an interesting story. An inverse relationship occurred between ambient temperatures and ${\dot{\text{V}}\text{O}}_{2}$, with the highest values occurring in the 4 °C condition. Interestingly, ${\dot{\text{V}}\text{O}}_{2}$ at 4 °C was 0.80 L min^−1^ higher than 21 °C at the 75 min time point despite a similar TTE. In addition, the 4 °C TTE had higher carbohydrate oxidation rates and respiratory exchange ratio at the 30 min time point, and the highest ${\dot{\text{V}}}_{{\text{E}}}$ rates throughout the TTE. The authors suggested that the lower temperatures might affect muscle metabolic or mechanical efficiency as discussed further below, and that because skin temperature was lowest at 4 °C ($T_{{{\text{Core}}}}$ was not different), skin thermal receptors perhaps increased ${\dot{\text{V}}}_{{\text{E}}}$ [34]. Ventilation is likely increased through reflex firing of afferent peripheral skin thermal receptors, non-myelinated nerve fibers, and perhaps vascular plexuses [51].

Cold’s effects on ${\dot{\text{V}}\text{O}}_{{2_{{{\text{Max}}}} }}$ are dependent on body temperature. In a classic study, Bergh and Ekblom studied the effects of cooling (oesophagus temperature) ranging from 38.4, 37.7, 35.8, 34.9 °C on ${\dot{\text{V}}\text{O}}_{{2_{{{\text{Max}}}} }}$, and of TTE in a severe domain work task [52]. Work time was the longest at 37.7 °C (6.8 min) and shortest at 34.9 °C (3.06 min) alongside the lowest reported ${\dot{\text{V}}\text{O}}_{{2_{{{\text{Peak}}}} }}$ (3.75 vs. 4.33 L min^−1^ at 37.7 °C). None of the subjects reached ${\dot{\text{V}}\text{O}}_{{2_{{{\text{Max}}}} }}$, when oesophageal and muscle temperature were lower than 37.5 and 38 °C respectively. This clearly demonstrates compromised work above CP with pre-cooling.

Conversely, in cross-country skiers performing a graded exercise test in − 15 °C and 23 °C, ${\dot{\text{V}}\text{O}}_{{2_{{{\text{Max}}}} }}$ was not different. However, at submaximal intensities,${\dot{\text{V}}\text{O}}_{2}$ and ${\dot{\text{V}}}_{{\text{E}}}$ were higher in − 15 °C but maximal ${\dot{\text{V}}}_{{\text{E}}}$ was lower at − 15 °C. No pre-cooling occurred nor was $T_{{{\text{Core}}}}$ measured, the skiers started the test upon entering the chamber, and were clothed adequately [53]. Likewise, cyclists completing a graded exercise test in either 30 °C or 10 °C (no precooling) showed no difference in ${\dot{\text{V}}\text{O}}_{{2_{{{\text{Max}}}} }}$. A lower lactate threshold in ambient versus cold conditions was reported, as were greater lactate levels at submaximal absolute workloads. Given lower skin temperatures in cold conditions (30.6 vs. 33.2 °C), cold-induced peripheral vasoconstriction could have had an effect on lactate [54]. Blood flow diverted from the skin could have been sent to inactive muscle for lactate metabolism, or permitted to reach the liver for conversion [54]. Discrepancies in results appear related to the protocol used before the exercise task: in other words, precooling of $T_{{{\text{Core}}}}$ and muscle appears necessary to affect performance.

Sprint efforts are also hindered by cold. Using the Wingate test, performance decreased following 30 min of waist-deep water immersion (~ 11 °C). Average and peak power output were 26% and 30% lower than control, suggesting that decreased contraction velocity as a result of the precooling is accountable for the decrements [55]. In a sprinting task, Bergh and Ekblom showed that in muscle cooled to 31.4 °C and $T_{{{\text{Core}}}}$ of 35.7 °C time-to-complete 20 pedal revolutions, maximal speed and initial power output were all lower than when the muscle was 38.3 °C alongside a $T_{{{\text{Core}}}}$ of 37.8 °C [56]. The authors discuss lower nerve conduction velocity and slowing of chemical reactions involved in cross bridges by the cold as potential mechanisms. Mechanistically, in animal preparations, it is appears that temperature increases the force and strain of cross-bridges, and as stated in a recent review, “*force is endothermic, and that force rises with temperature, upon absorption of heat. This is largely due to the force generation by an attached crossbridge state itself being temperature-sensitive*” [57, 58]. As such, precooling hinders sarcomere performance, thus affecting sprint type work and consequently *W*′.

Studies have also examined upper limb sprint performance. Cross-country skiers performed two sets of double poling sprint efforts of 30 and 120 s followed by an incremental test to failure in either − 14 °C or 6 °C over a 54 min exposure, without precooling. No significant difference occurred in power output during the first sprints early in the exposure, but the second sprints in − 14 °C later in the exposure had lower power output compared to 6 °C. Additionally, body and skin temperature were lower at the end of the − 14 °C trial and ${\dot{\text{V}}\text{O}}_{2}$, heart rate and maximal power output were lower during the end of the incremental test, despite no difference in RPE [59]. Single muscles also experience decreased force performance in the cold. Thumb adductors cooled to 22 °C for 20 min show a ~ 79% decreased force production compared to 37 °C [60].

Various mechanisms explain the negative effects of cold. Aerobically speaking, we have known since 1909 that low temperatures shift the hemoglobin dissociation curve left, thus consequently lowering oxygen offloading at the tissues [61], while enhancing uptake at the lung which can be particularly important in cold, hypoxic environments [62]. Further, Castellani and Tipton summarise three key factors affecting aerobic performance in the cold as: (1) decreased core, muscle and skin temperature (2) altered metabolic processes, such as a greater reliance on anaerobic metabolism as seen by greater blood and muscle lactate values, and decreased efficiency in cold versus ambient temperature exercise tasks [63] and (3) circulatory impairments including decreased ${\text{HR}}_{{{\text{Max}}}}$, cardiac output, and muscle blood flow [63]. As discussed in the skier study, cellular mechanisms contribute to the decrements in sprint performance [59]. Laboratory studies confirm that cold affects muscle contraction velocity. For example, cooled mouse skeletal muscle shows decreased velocity of shortening and lengthening. A proposed explanation for the decreased shortening would be cold’s effect on actomyosin ATPase, whereas the longer lengthening cycle may be caused by decreased cross bridge detachment rates [59, 64]. As with heat, it is worthwhile to highlight that the common theme is that core temperature changes or local cooling of a specific muscle group are causative, and not simply the cold environment itself. In summary, these decrements in sprint and maximal aerobic capacity indicate that both CP and *W*′ are decreased by cold when sufficient pre-cooling of the muscle and/or $T_{{{\text{Core}}}}$ occur.

### Air Pollution

Outdoor urban sporting activities are increasingly subject to air pollution. Exercise at CP requires substantial ${\dot{\text{V}}}_{{\text{E}}}$, and since ${\dot{\text{V}}}_{{\text{E}}}$ increases with exercise intensity, exposure to pollutants concomitantly rises [65] especially with the shift to predominantly oral breathing as exercise intensity increases [66]. Pollution is a combination of gases (such as ozone) and particulate matter (PM). PM is produced from human (i.e. combustion of fuels) or natural sources (i.e. forest fires) and are a mix of liquid and solid and vary in size ranging from coarse (2.5–10 µm), fine (≤ 2.5 µm) to ultrafine (≤ 0.1 µm) [65].

Observational studies with ozone (O_3_) most consistently show detrimental effects, whereas traffic related air pollutants and particulates are not consistently linked to impaired performance. Physiologically, O_3_ is a highly reactive lung irritant that causes oedema of respiratory epithelial surfaces leading to dyspnea, impaired pulmonary function (higher frequency of breathing, lower tidal volume, impaired FEV_1_) and heart rate variability while increasing perceived exertion [65, 67, 68]. A plausible mechanism responsible for the detrimental effect of O_3_ is likely due to the formation of reactive oxygen metabolites and “*ozononation of fatty acids present at epithelial cell surfaces and in lung lining fluids*” [69]. One group examined 5000 m times in professional runners (Diamond League) in conjunction with meteorological conditions across multiple cities. The slowest city, Birmingham, had the highest O_3_. Conversely, Paris (the fastest city), had the lowest O_3_ levels. Not surprisingly, higher temperatures and wind also affected performance [70]. Another retrospective analysis observed detrimental effects of O_3_ on “aerobic” events in ~ 1700 track and field meets. A 0.39% performance detriment was attributed for every 10 ppb rise in O_3_ [71]. In terms of PM effects, an observational study of marathon running showed that increases in PM were correlated to slower times, albeit only in women [72].

Well-controlled laboratory trials have also been performed. Cycling slightly below CP (60%${\dot{\text{V}}\text{O}}_{{2_{{{\text{Max}}}} }}$) in recreationally active subjects for 30 min in diesel exhaust yields higher RPE than in filtered air with minimal differences in cardiorespiratory, vascular and performance parameters [73, 74]. Of the few studies showing a detrimental effect of PM, subjects were exposed in a crossover design to either low or high PM for 20 min while cycling at 60% ${\text{HR}}_{{{\text{Max}}}}$, immediately followed by a 6 min time trial. Work done was lower in high PM (108.0 ± 14.8 vs. 104.9 ± 15.2 kJ). Decreased flow-mediated dilation was deemed partially responsible for the decrement, as it could potentially decrease blood flow to the exercising muscle [75].

Researchers have also examined controlled exposures to ambient air pollution. However, these studies can be challenging to interpret given the lack of blinding and the predominant use of indirect measures. For example, predicted ${\dot{\text{V}}\text{O}}_{{2_{{{\text{Max}}}} }}$ (shuttle run) was lower in trained and untrained individuals performing the test in a high versus low-pollution area [76]. Interestingly, they measured a lowered red blood cell count and hematocrit in the high-pollution area, whereas white blood cell and platelet count increased from pre-exercise. Additionally, ${\text{HR}}_{{{\text{Max}}}}$ was greater in high pollution in the untrained group only. Given the detrimental haematological results, the authors propose a decreased oxygen carrying capacity which ultimately lessened ${\dot{\text{V}}\text{O}}_{{2_{{{\text{Max}}}} }}$ [76]. Conversely, two studies by Wagner et al. [77, 78] looked at performance in cycling (20 min) and running (~ 14 min) time trials, and found no difference in time trial performance, indicating negligible effects on CP/CS. In contrast, a study comparing exercise response in firefighters did not detect a decrease in ${\dot{\text{V}}\text{O}}_{{2_{{{\text{Max}}}} }}$ in a high pollution environment; however GET and heart rate and ${\dot{\text{V}}\text{O}}_{2}$ at GET were lower in pollution [79]. The reader may further examine the effects of air pollution on acute exercise in this recent systematic review [80].

Sprint studies in air pollution are sparse. A study in Chinese Super League Soccer analysing 240 matches showed that air quality index did not affect technical skills (i.e. passes) but influenced sprint distance (distance covered when travelling ≥ 23 km h^−1^) [81]. We can infer that in this scenario air pollution had an impact on *D*’ the distance travelled (m) above critical speed [1] which in this study was impaired by environmental factors.

In summary, the picture is quite mixed due to the varying study designs and pollution conditions (PM size, dosage, O_3_). However, it seems clear that ozone exposure raises RPE, impairs respiratory function and induces airway irritation [67], while PM may alter vascular function. Therefore, in healthy individuals, PM effects on CP are uncertain, while O_3_ effects are more clear, and would likely lead to a lowering of CP. *W*′ is potentially affected; however additional evidence is required.

## Conclusion

As examined, environmental stressors predominantly lower performance at the severe domain boundary, and given sufficient exposure, at higher intensities as well (Table 1). Hypoxia lowers ambient ${\text{P}}_{{{\text{I}}_{{{\text{O}}_{2} }} }}$, impairing the cardiovascular system’s ability to deliver sufficient oxygen and thus lowering CP and ${\dot{\text{V}}\text{O}}_{{2_{{{\text{Max}}}} }}$. *W*′ is only affected in severe hypoxia. In heat, cardiovascular decrements affect performance if $T_{{{\text{Core}}}}$ is sufficiently raised whereas short supra-CP bouts are unaffected. However, with pre-heating or several consecutive sprint bouts, a performance decrement is likely. At the other extreme, performance in cold without pre-cooling does not appear to harm work near the severe domain, if the workload is sufficiently high to maintain $T_{{{\text{Core}}}}$ via metabolic heat production. Sprint performance with pre-cooling is considerably affected. Given the heterogeneous composition of air pollution, the picture is less clear. Ozone increases symptoms and reduces running speeds, while particulate dominant air pollution has less of a consistent effect near CP. Given this review solely focused on acute effects of each of the environmental stressors, further work should consider the effects of chronic exposures and especially of acclimatization to these environments on CP and *W*′. The various ways by which environmental extremes influence exercise at CP highlight the array of integrated systems which enable exercise performance. Those aiming to optimize pacing for competition or train in adverse environmental conditions should consider the individual stressor's effect on CP and *W*′.

**Table 1:** Effect of environmental condition on critical power and *W'*

	Environmental condition			
	Acute hypoxia	Heat	Cold	Air pollution
Effects on critical power	↓	↓	↓	↓/ ↔
Effects on *W*′	↓ In severe hypoxia	↓ If consecutive sprints or prewarmed	↓ If precooled	?

↓ = decrease, ↔  = no change, ? = unclear

## Data Availability

Not applicable.
